# Supplementary material for: Landscape Heterogeneity Drives Genetic Diversity in the Highly Dispersive Moss Funaria hygrometrica Hedw
Source: Plants (Basel). 2024 Oct 4;13(19):2785. doi: 10.3390/plants13192785 (PMC11478527; doi:10.3390/plants13192785)
Supplement: Supplementary file 1 [file plants-13-02785-s001.zip › plants-3177291-supplementary.pdf]

Supplementary Data

Table S1: Significance Test Results for π-values in Sierra Nevada Mountains (SN) and Murcia Region (MU). This table presents the results of the significance test conducted on the different π-values observed in Sierra Nevada Mountains (SN) and Murcia Region (MU). A total of 10,000 bootstrap replicates of the sequences were generated, and the π-values of these replicates were calculated. The table includes the mean values of the replicates, their standard deviation, and the corresponding 95% confidence intervals.

Region	mean (π)	Standard Deviation	Quantile	
			2.5%	97.5%
**ITS1-SN**	0.00522	0.00156	0.00251	0.00852
**ITS1-MU**	0.00129	0.00057	0.00020	0.00239
**ITS1**	0.00411	0.00113	0.00214	0.00652
**ITS2-SN**	0.00863	0.00215	0.00452	0.01283
**ITS2-MU**	0.00407	0.00217	0.00000	0.00843
**ITS2**	0.00750	0.00175	0.00422	0.01104
**cpDNA-SN**	0.00198	0.00026	0.00146	0.00251
**cpDNA-MU**	0.00023	0.00021	0.00000	0.00065
**cpDNA**	0.00166	0.00023	0.00122	0.00212
**mtDNA-SN**	0.00038	0.00014	0.00012	0.00070
**mtDNA-MU**	0.00000	0.00000	0.00000	0.00000
**mtDNA**	0.00023	0.000009	0.00007	0.00044

Table S2: Information on Collected Samples. This table provides details about the collected material, including the region, nearest city, location information (code, description, geographical coordinates, height in meters above sea level), sample code, MUB herbarium reference, and registration number of four genotyped loci (accessions of deposited haplotypes in GenBank along with the number of haplotypes).

Region	Nearest city	Location code, description (geographical coordinates and height in m a.s.l.)	Sample code	Accessions				
				MUB	ITS1 (Hap no.)	ITS2 (Hap no.)	cpDNA (Hap no.)	mtDNA (Hap no.)
Sierra Nevada – Zone 1 (SN-Z1)	Motril	**SN-Z1**.1: Road N-340 from El-Ejido to Motril, km 330 (36° 43' 57.4" N; 03° 31' 00.9" W; 10)	SN_01	43989	JX985532 (1)	JX985554 (1)	JX985507 (1)	JX912563 (1)
		**SN-Z1**.2: Road N-from El-Ejido to Motril, km 334 (36° 43' 47.4" N; 03° 30' 30.8" W; 10)	SN_02	43991	JX985533 (2)	JX985555 (2)	JX985508 (2)	JX912564 (2)
			SN_03	43993	JX985533 (2)	JX985555 (2)	JX985508 (2)	JX912564 (2)
			SN_04	43995	JX985533 (2)	JX985555 (2)	JX985508 (2)	JX912564 (2)
		**SN-Z1**.3: Road N-340 from El-Ejido to Motril , km 370 (36° 45' 4.60" N; 03° 11' 59.6" W; 51)	SN_05	43999	JX985534 (3)	JX985554 (1)	JX985507 (1)	JX912563 (1)
			SN_06	44001	JX985535 (4)	JX985554 (1)	JX985509 (5)	JX912563 (1)
	Órgiva	**SN-Z1**.4: Road A-346 from Vélez de Benaudalla to Órgiva, km 9 (36° 51' 56.5" N; 03° 28' 59.6" W; 288)	SN_07	44005	JX985534 (3)	JX985554 (1)	JX985507 (1)	JX912563 (1)
			SN_08	44023	JX985534 (3)	JX985554 (1)	JX985510 (4)	JX912563 (1)
			SN_09	44024	JX985534 (3)	JX985554 (1)	JX985507 (1)	JX912565 (3)
		**SN-Z1**.5: Road A-346 from Vélez de Benaudalla to Órgiva, km 7 (36° 52' 19.8" N; 03° 28' 24.3" W; 384)	SN_10	44026	JX985536 (5)	JX985554 (1)	JX985507 (1)	JX912563 (1)
			SN_11	44027	JX985537 (6)	JX985554 (1)	JX985507 (1)	JX912563 (1)
			SN_12	44034	JX985538 (7)	JX985554 (1)	JX985507 (1)	JX912563 (1)
			SN_13	44035	JX985539 (8)	JX985554 (1)	JX985507 (1)	JX912563 (1)
			SN_14	44036	JX985539 (8)	JX985554 (1)	-	JX912563 (1)
			SN_15	44037	JX985534 (3)	JX985554 (1)	-	JX912563 (1)
			SN_16	44038	JX985539 (8)	JX985554 (1)	JX985511 (5)	JX912563 (1)
			SN_17	44039	JX985534 (3)	JX985554 (1)	-	JX912563 (1)
	Lanjarón	**SN-Z1**.6: Lanjarón city (36° 55' 1.60" N; 03° 28' 47.9" W; 638)	SN_18	44040	JX985534 (3)	JX985554 (1)	JX985507 (1)	JX912563 (1)
			SN_19	44041	JX985534 (3)	JX985554 (1)	JX985507 (1)	JX912563 (1)
			SN_20	44042	JX985534 (3)	JX985554 (1)	JX985512 (6)	JX912563 (1)
			SN_21	44044	JX985534 (3)	JX985554 (1)	JX985507 (1)	JX912563 (1)

		**SN-Z1**.7: Out of Lanjarón city, in the road A-348 to Órgiva (36° 55' 1.40" N; 03° 28' 17.0" W; 647)	SN_22	44051	JX985540 (9)	JX985556 (3)	JX985513 (7)	JX912564 (2)
			SN_23	44054	JX985540 (9)	JX985557 (4)	JX985513 (7)	JX912564 (2)
			SN_24	44060	JX985540 (9)	JX985556 (3)	JX985513 (7)	JX912564 (2)
Sierra Nevada – Zone 2 (SN-Z2)	Cenés de la Vega	**SN-Z2**.1: Cenés de la Vega surroundings (37° 09' 17.4" N; 03° 32' 46.2" W; 755)	SN_25	44061	JX985541 (10)	JX985554 (1)	-	JX912563 (1)
			SN_26	44062	JX985534 (3)	JX985554 (1)	JX985514	JX912563 (1)
			SN_27	44063	JX985542 (11)	JX985554 (1)	JX985515 (9)	JX912563 (1)
			SN_28	44064	JX985543 (12)	JX985554 (1)	JX985507 (1)	JX912563 (1)
			SN_29	44065	JX985534 (3)	JX985554 (1)	JX985516 (10)	JX912563 (1)
			SN_30	44066	JX985534 (3)	JX985554 (1)	JX985517 (11)	JX912563 (1)
			SN_31	44067	JX985537 (6)	JX985554 (1)	JX985507 (1)	JX912563 (1)
			SN_32	44070	JX985544 (13)	JX985554 (1)	JX985518 (12)	JX912563 (1)
		**SN-Z2**.2: Road GR-420 from Cenés de la Vega to Pinos Genil, km 1 (37° 09' 41.9" N; 03° 31' 27.2" W; 750)	SN_33	44071	JX985534 (3)	JX985554 (1)	JX985519 (13)	JX912563 (1)
			SN_34	44074	JX985534 (3)	JX985554 (1)	JX985520 (14)	JX912563 (1)
			SN_35	44075	JX985545 (14)	JX985554 (1)	JX985507 (1)	JX912563 (1)
			SN_36	44076	JX985534 (3)	JX985554 (1)	JX985507 (1)	JX912563 (1)
			SN_37	44077	JX985540 (9)	JX985556 (3)	JX985521 (15)	JX912564 (2)
			SN_38	44078	JX985546 (15)	JX985554 (1)	-	JX912563 (1)
			SN_39	44079	JX985534 (3)	JX985554 (1)	JX985519 (13)	JX912563 (1)
			SN_40	44081	JX985547 (16)	JX985554 (1)	JX985507 (1)	JX912563 (1)
			SN_41	44082	JX985534 (3)	JX985554 (1)	JX985507 (1)	JX912563 (1)
			SN_42	44083	JX985545 (14)	JX985554 (1)	JX985507 (1)	JX912563 (1)
	Canales	**SN-Z2**.3: Road A-395 from Granada city to Sierra Nevada, km 15 (37° 08' 34.1" N; 03° 29' 14.5" W; 1294)	SN_43	44085	JX985534 (3)	JX985554 (1)	JX985522 (16)	JX912563 (1)
			SN_44	44086	JX985537 (6)	JX985554 (1)	JX985516 (10)	JX912563 (1)
			SN_45	44087	JX985534 (3)	JX985554 (1)	JX985507 (1)	JX912563 (1)
			SN_46	44088	JX985536 (5)	JX985554 (1)	JX985523 (17)	JX912563 (1)
			SN_47	44089	JX985546 (15)	JX985554 (1)	JX985507 (1)	JX912563 (1)
			SN_48	44090	JX985534 (3)	JX985554 (1)	JX985507 (1)	JX912563 (1)
			SN_49	44091	JX985532 (1)	JX985554 (1)	JX985524 (18)	JX912563 (1)
			SN_50	44092	JX985534 (3)	JX985554 (1)	JX985525 (19)	JX912563 (1)
			SN_51	44093	JX985548 (17)	JX985554 (1)	JX985507 (1)	JX912563 (1)
			SN_52	44094	JX985534 (3)	JX985554 (1)	JX985507 (1)	JX912569 (7)
			SN_53	44095	JX985534 (3)	JX985554 (1)	JX985507 (1)	JX912563 (1)

		**SN-Z2**.4: Road A-395 from Granada city to Sierra Nevada, km 16 (37° 08' 22.7" N; 03° 29' 03.5" W; 1328)	SN_54	44096	JX985534 (3)	JX985554 (1)	JX985507 (1)	JX912563 (1)
			SN_55	44097	JX985546 (15)	JX985554 (1)	JX985507 (1)	JX912563 (1)
			SN_56	44098	JX985534 (3)	JX985554 (1)	JX985507 (1)	JX912563 (1)
			SN_57	44100	JX985534 (3)	JX985554 (1)	JX985507 (1)	JX912563 (1)
			SN_58	44102	JX985534 (3)	JX985554 (1)	JX985507 (1)	JX912563 (1)
			SN_59	44103	JX985534 (3)	JX985554 (1)	JX985507 (1)	JX912563 (1)
			SN_60	44105	JX985540 (9)	JX985554 (1)	JX985517 (11)	JX912563 (1)
			SN_61	44109	JX985549 (18)	JX985554 (1)	JX985507 (1)	JX912563 (1)
			SN_62	44110	JX985546 (15)	JX985554 (1)	JX985507 (1)	JX912563 (1)
Sierra Nevada – Zone 3 (SN-Z3)	Güejar Sierra	**SN-Z3**.1: Road A-395, from Granada city to Sierra Nevada, barranco de las Víboras (37° 07' 58.0" N; 03° 26' 20.6" W; 1644)	SN_63	44111	JX985551 (20)	JX985558 (5)	JX985507 (1)	JX912563 (1)
			SN_64	44112	JX985536 (5)	JX985554 (1)	JX985507 (1)	JX912563 (1)
			SN_65	44113	JX985534 (3)	JX985554 (1)	JX985526 (20)	JX912564 (2)
			SN_66	44115	JX985540 (9)	JX985556 (3)	JX985526 (20)	JX912567 (5)
			SN_67	44116	JX985534 (3)	JX985554 (1)	JX985526 (20)	JX912568 (6)
			SN_68	44119	JX985540 (9)	JX985556 (3)	JX985526 (20)	JX912564 (2)
			SN_69	44120	JX985534 (3)	JX985559 (6)	JX985527 (21)	JX912563 (1)
			SN_70	44121	JX985550 (19)	JX985560 (7)	JX985526 (20)	JX912564 (2)
		**SN-Z3**.2: Road A-395 from Granada city to Sierra Nevada, center for nature interpretation El Dornajo (37° 07' 57.9" N; 03° 26' 06.2" W; 1666)	SN_71	44123	JX985534 (3)	JX985554 (1)	JX985507 (1)	JX912563 (1)
			SN_72	44124	JX985534 (3)	JX985554 (1)	JX985507 (1)	JX912566 (4)
			SN_73	44125	JX985540 (9)	JX985554 (1)	JX985528 (22)	JX912563 (1)
			SN_74	44127	JX985534 (3)	JX985554 (1)	JX985529 (23)	JX912563 (1)
			SN_75	44128	JX985552 (21)	JX985554 (1)	JX985507 (1)	JX912570 (8)
			SN_76	44129	JX985540 (9)	JX985554 (1)	JX985507 (1)	JX912563 (1)
Sierra Nevada – Zone 4 (SN-Z4)	Ski Station	**SN-Z4**.1: Ski resort Pradollano (37° 05' 35.7" N; 03° 23' 54.5" W; 2156)	SN_77	44131	JX985534 (3)	JX985555 (2)	JX985507 (1)	JX912563 (1)
			SN_78	44132	JX985537 (6)	JX985555 (2)	JX985530 (24)	JX912571 (9)
		**SN-Z4**.2: Road A-395 from Granada city to Sierra Nevada, military mountain hostel (37° 06' 47.9" N; 03° 25' 10" W; 2210)	SN_79	44139	JX985534 (3)	JX985555 (2)	JX985507 (1)	JX912572 (10)
			SN_80	44140	JX985553 (22)	JX985555 (2)	JX985531 (25)	JX912573 (11)
		**SN-Z4**.3: Ski resort Borreguiles, artificial lake (37° 04' 22.3" N; 03° 23' 28.2" W; 2623)	SN_81	44141	JX985540 (9)	JX985554 (1)	JX985508 (2)	JX912564 (2)
			SN_82	44142	JX985540 (9)	JX985556 (3)	JX985508 (2)	JX912564 (2)
			SN_83	44143	JX985546 (15)	JX985554 (1)	JX985508 (2)	JX912563 (1)

		**SN-Z4**.4: Ski resort Borreguiles (37° 04' 16.4" N; 03° 23' 14.6" W; 2683)	SN_84	44148	JX985540 (9)	JX985556 (3)	JX985507 (1)	JX912564 (2)
Murcia – Area 1 (MU-A1)	Totana	**MU-A1**.1.1: Transfer canal road between Totana and Alhama de Murcia (1) (37° 48' 59.5" N; 01° 29' 33.9" W; 339)	MU_01	43926	JX985534 (3)	JX985554 (1)	-	KX387646 (12)
			MU_02	43927	JX985534 (3)	-	-	KX387646 (12)
			MU_03	43928	JX985534 (3)	JX985554 (1)	-	KX387646 (12)
			MU_04	43929	KX387636 (23)	JX985554 (1)	-	KX387646 (12)
		**MU-A1**.1.2: Transfer canal road between Totana and Alhama de Murcia (2) (37° 49' 29.0" N; 01° 29' 21.8" W; 342)	MU_05	43930	KX387636 (23)	JX985554 (1)	-	KX387646 (12)
			MU_06	43931	JX985534 (3)	JX985554 (1)	JX985507 (1)	KX387646 (12)
			MU_07	43932	JX985534 (3)	JX985554 (1)	JX985507 (1)	KX387646 (12)
			MU_08	43933	-	-	JX985507 (1)	-
			MU_09	43934	JX985534 (3)	JX985554 (1)	-	KX387646 (12)
			MU_10	43935	-	-	-	KX387646 (12)
			MU_11	43936	JX985534 (3)	JX985554 (1)	-	KX387646 (12)
			MU_12	43937	-	-	-	KX387647 (13)
			MU_13	43938	JX985534 (3)	JX985554 (1)	-	KX387646 (12)
	Alhama de Murcia	**MU-A1**.2.1a: Outside the industrial area of Alhama de Murcia (37° 49' 19.0" N; 01° 24' 11.5" W; 157)	MU_14	43896	JX985534 (3)	JX985554 (1)	KX387643 (27)	KX387646 (12)
		**MU-A1**.2.1b: Outside the industrial area of Alhama de Murcia (37°51' 35.8" N; 01° 24' 02.1" W; 183)	MU_15	43921	JX985534 (3)	JX985554 (1)	-	KX387646 (12)
			MU_16	43922	KX387637 (24)	JX985554 (1)	JX985507 (1)	KX387646 (12)
			MU_17	43923	-	-	-	KX387646 (12)
			MU_18	43924	JX985534 (3)	JX985554 (1)	-	KX387646 (12)
			MU_19	43925	JX985534 (3)	JX985554 (1)	-	KX387646 (12)
		**MU-A1**.2.2: A7 freeway, connexion with Alhama de Murcia (37° 51' 35.8" N; 01° 24' 02.1" W; 183)	MU_20	43915	-	-	-	KX387646 (12)
		**MU-A1**.2.4: Transfer canal road between Totana and Alhama de Murcia (3) (37° 51' 10.9" N; 01° 28' 09.0" W; 343)	MU_21	43940	-	-	-	KX387646 (12)
Murcia	La Unión	**MU-A2**.1.1: Atamaría, Rambla de la Carrasquilla (37° 35' 42.2" N; 00° 49' 25.8" W; 139)	MU_22	43895	JX985534 (3)	JX985554 (1)	KX387642 (26)	KX387646 (12)

	**MU-A2**.1.2: El Gorguel, Cartagena-La Unión Mining District, W slope (37° 35' 35.1" N; 00° 52' 51.6" W; 120)	MU_23	43902	JX985534 (3)	JX985554 (1)	JX985507 (1)	KX387646 (12)
	**MU-A2**.1.3: El Gorguel, Cartagena-La Unión Mining District, E slope (37° 35' 30.8" N; 00° 52' 45.9" W; 108)	MU_24	43903	-	-	-	KX387646 (12)
Cartagena	**MU-A2**.2.1: Road RM-F54, from Los Alcázares to Los Urrutias) (37° 42' 17.5" N; 00° 51' 00.0" W; 002)	MU_25	43891	-	-	-	KX387646 (12)
	**MU-A2**.2.2: Los Nietos, at the road RM-F54, (37° 39' 05.8" N; 00° 47' 39.5" W; 004)	MU_26	43892	JX985534 (3)	JX985554 (1)	-	KX387646 (12)
		MU_27	43901	JX985534 (3)	JX985554 (1)	KX387645 (29)	KX387646 (12)
		MU_28	439xx	JX985534 (3)	JX985554 (1)	-	KX387646 (12)
	**MU-A2**.2.3: Cala Reona (37° 37' 05.6" N; 0° 42' 54.1" W; 5)	MU_29	43941	JX985534 (3)	JX985554 (1)	-	KX387646 (12)
	**MU-A2**.2.4: Boca Rambla, Venta Simón (37° 43' 22.0" N; 0° 51' 41.8" W; 3)	MU_30	43942	-	-	JX985507 (1)	KX387646 (12)
San Javier	**MU-A2**.3.1a: Los Narejos village (1) (37° 45' 26.7" N; 0° 51' 01.4" W; 7)	MU_31	43894	JX985534 (3)	JX985554 (1)	JX985507 (1)	KX387646 (12)
	**MU-A2**.3.1b: Los Narejos village (2) (37° 45' 27.5" N; 0° 51' 01.4" W; 7)	MU_32	43904	JX985534 (3)	JX985554 (1)	JX985507 (1)	KX387646 (12)
		MU_33	43905	JX985534 (3)	JX985554 (1)	JX985507 (1)	KX387646 (12)
		MU_34	43906	KX387636 (23)	JX985554 (1)	JX985507 (1)	KX387646 (12)
		MU_35	43907	JX985534 (3)	JX985554 (1)	-	KX387646 (12)
		MU_36	43908	JX985534 (3)	JX985554 (1)	JX985507 (1)	KX387646 (12)
		MU_37	43909	-	-	-	KX387646 (12)
		MU_38	43910	JX985534 (3)	JX985554 (1)	JX985507 (1)	KX387646 (12)
		MU_39	43911	JX985534 (3)	-	-	KX387646 (12)
		MU_40	43912	JX985534 (3)	JX985554 (1)	-	KX387646 (12)
		MU_41	43913	-	-	-	KX387646 (12)
		MU_42	43914	KX387637 (24)	JX985554 (1)	-	KX387646 (12)
	**MU-A2**.3.2: Lo Pagán, San Pedro del Pinatar, public garden "Parque del Mar" (37° 48' 57.3" N; 0° 47' 6.84" W; 2)	MU_43	43943	-	-	JX985507 (1)	KX387646 (12)
		MU_44	43944	JX985534 (3)	JX985554 (1)	-	KX387646 (12)

Murcia – Area 3 (MU-A3)	Las Torres de Cotillas	**MU-A3**.1.1: Las Torres de Cotillas village (38° 00' 56.0" N; 01° 14' 44.0" W; 101)	MU_45	43897	JX985534 (3)	KX387639 (8)	JX985507 (1)	KX387646 (12)
			MU_46	43917	KX387637 (24)	KX387641 (10)	-	KX387646 (12)
		**MU-A3**.1.2: Industrial area at Media Legua, Las Torres de Cotillas (38° 00' 49.1" N; 01° 13' 31.3" W; 83)	MU_47	43918	JX985534 (3)	JX985554 (1)	JX985507 (1)	KX387646 (12)
			MU_48	43919	JX985534 (3)	JX985554 (1)	-	KX387646 (12)
			MU_49	43920	JX985534 (3)	JX985554 (1)	-	KX387646 (12)
	Fortuna	**MU-A3**.2.1: Fortuna, Rambla Salada (38° 04' 38.4" N; 01° 11' 52.0" W; 115)	MU_50	43899	-	-	-	KX387646 (12)
	Blanca	**MU-A3**.3.1: Near El Rellano, in the road RM-A20 coming from Estación de Blanca (38° 12' 45.6" N; 01° 14' 13.1" W; 307)	MU_51	43900	KX387638 (25)	KX387640 (9)	KX387644 (28)	KX387646 (12)
	Abanilla	**MU-A3**.4.1: Abanilla field (38° 14' 03.3" N; 01° 02' 30.7" W; 212)	MU_52	43898	KX387636 (23)	JX985554 (1)	JX985507 (1)	KX387646 (12)
	Murcia	**MU-A3**.5.1: Javalí Viejo, milatiry base (37° 59' 25.5" N; 01° 13' 10.4" W; 77)	MU_53	43916	JX985534 (3)	JX985554 (1)	JX985507 (1)	KX387646 (12)

Table S3: Primers used for amplification and DNA sequencing. Each primer includes the locus, length (in base pairs), primer name, sequences, and annealing temperature. Any altered or newly introduced nucleotides compared to the reference sequences are highlighted in bold.

Locus	Length (bp)	Primer name	5´-Sequence-3´	Annealing (ºC)
ITS1	28	101-F-bryG	**C**CGA**T**T**GA**ATGGTCCGGTGA**G**GT**T**TTCG	60
	20	5.8s-R	GCTGCGTTCTTCATCGTTGC	
ITS2	20	5.8s-F	GCAACGATGAAGAACGCAGC	65
	31	102-R-bryN	**GCTGGGCTC**T**TT**CCGGTTCGCTCGCCGTTAC	
Funaria *rps*3-*rpl*16	22	*rps*3-F1-Fun	**CCAGCTCAAACAATTTATGGAG**	50
	24	*rpl*16-R1-Fun	**CATTCTCCCTCTATGTTGTTTACG**	
	17	*rpl*16-R2-Fun	**GAATTACCTCGGGTAGC**	
	19	*rpl*16-R3-Fun	**AAGCAATAGAATTACCTCG**	
*rpl*5-*rpl*16	18	*rpl*16-F1	GGATGGTGTGAGTTTGTC	54
	18	*rpl*5-R1	CGGAGTCTATTTGGAGTG	

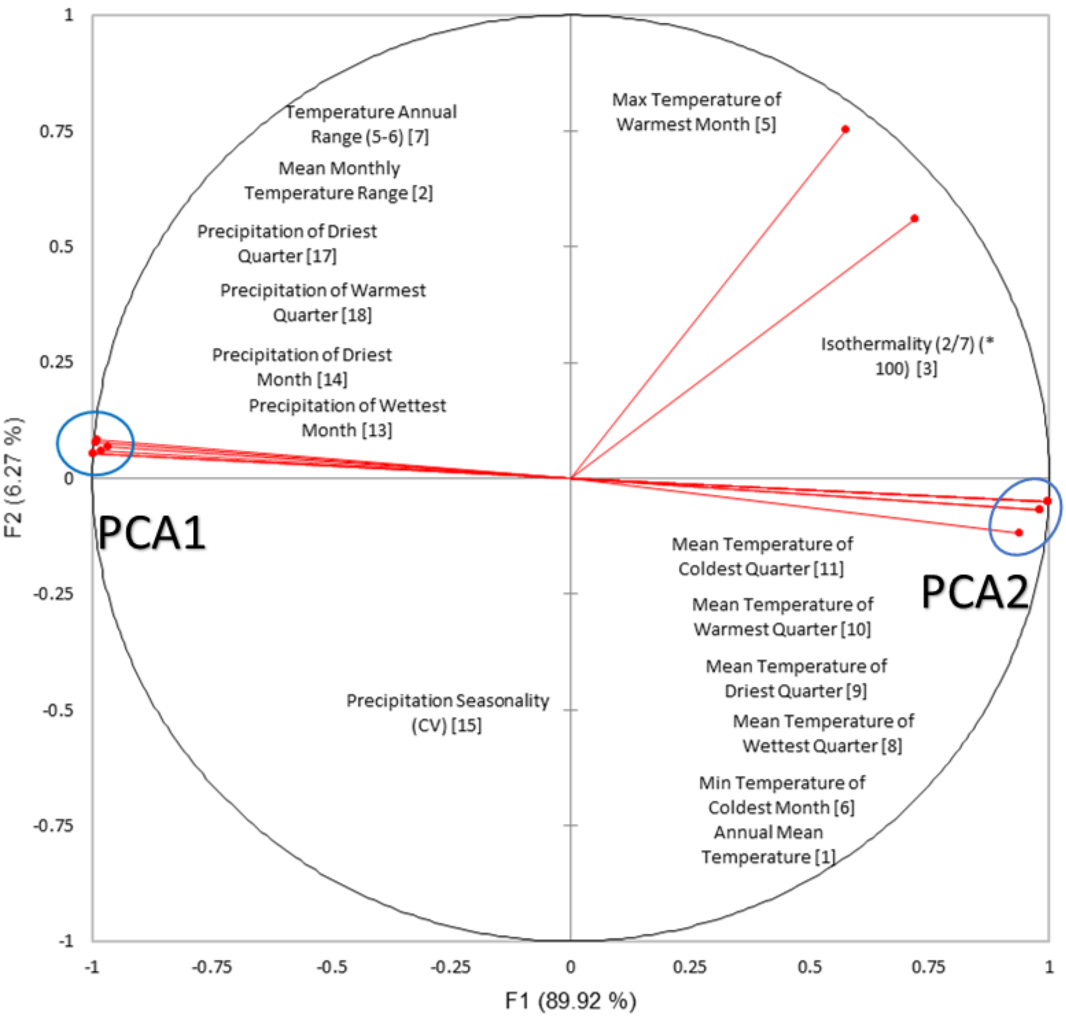

Figure S1: PCA plot for 19 bioclimatic variables explained by two axes. Axis F1 = 89.92 % and axis F2 = 6.27 %. Two PCA groups and two singletons were defined.
